# Use of Space–Time Models to Investigate the Stability of Patterns of Disease

**DOI:** 10.1289/ehp.10814

**Published:** 2008-04-25

**Authors:** Juan Jose Abellan, Sylvia Richardson, Nicky Best

**Affiliations:** 1 Small Area Health Statistics Unit, Department of Epidemiology and Public Health, Imperial College London, London, United Kingdom; 2 CIBER Epidemiología y Salud Pública, Spain; 3 Department of Epidemiology and Public Health, Imperial College London, London, United Kingdom

**Keywords:** Bayesian hierarchical models, congenital anomalies, disease mapping, mixture models, space-time interactions, stable disease patterns

## Abstract

**Background:**

The use of Bayesian hierarchical spatial models has become widespread in disease mapping and ecologic studies of health–environment associations. In this type of study, the data are typically aggregated over an extensive time period, thus neglecting the time dimension. The output of purely spatial disease mapping studies is therefore the average spatial pattern of risk over the period analyzed, but the results do not inform about, for example, whether a high average risk was sustained over time or changed over time.

**Objective:**

We investigated how including the time dimension in disease-mapping models strengthens the epidemiologic interpretation of the overall pattern of risk.

**Methods:**

We discuss a class of Bayesian hierarchical models that simultaneously characterize and estimate the stable spatial and temporal patterns as well as departures from these stable components. We show how useful rules for classifying areas as stable can be constructed based on the posterior distribution of the space–time interactions. We carry out a simulation study to investigate the sensitivity and specificity of the decision rules we propose, and we illustrate our approach in a case study of congenital anomalies in England.

**Results:**

Our results confirm that extending hierarchical disease-mapping models to models that simultaneously consider space and time leads to a number of benefits in terms of interpretation and potential for detection of localized excesses.

Geographic epidemiology and public health surveillance have benefited from combined advances in the statistical modeling of spatial data and in geographic information systems. Exploring and characterizing a variety of spatial patterns of diseases at a fine geographic resolution have become possible (Banerjee et al. 2004), and the use of hierarchical models estimated in a Bayesian framework to account for different levels of variability of such data is now well established (Richardson 2003). Inference on the relative risks of interest is usually obtained through the implementation of Bayesian computations that output the posterior distribution of the relative risks in each area. On the basis of these posterior distributions, Richardson et al. (2004) have calibrated decision rules to detect areas of increased risks. Insight into the sensitivity of the resulting inference to the choice of the structure of the different components of the hierarchical model has been gained through the use of simulation studies (Best et al. 2005) and numerous case studies (see, e.g., Ferrándiz et al. 2002; Jarup et al. 2002; Pascutto et al. 2000; Ramis-Prieto et al. 2007).

Most studies consider data aggregated over a period of time, so they cannot address important epidemiologic questions about the stability of the estimated spatial patterns of disease. Indeed, two quite different situations can give rise to the same accumulated number of cases in an area over a set time period: *a*) the rate of accumulation in any subinterval of time is constant or varies slowly, with the same pattern of variation for all areas, or *b*) the rate of accumulation over time has substantial and distinctive variability for that area. The epidemiologic interpretations of these two situations are quite different. Spatial patterns corresponding to situation *a*) occur in a “constant manner” over time and hence could be induced by environmental or sociodemographic risk factors that act in a sustained way throughout the whole period. In contrast, situation *b*) will lead to substantial variability of the pattern of risk over time, pointing to potentially emerging short-latency risk factors that would create a high excess of cases in a few short time intervals or, alternatively, to artifactual variations possibly due to abrupt changes in recording practices in some areas. Hence, uncovering the full space–time profile of the risks would considerably strengthen the epidemiologic interpretation of overall patterns of risk, and particularly of high-risk areas. This leads naturally to the use of space–time models for analyzing small-area disease variability. These models characterize predictable spatial and temporal patterns and simultaneously estimate specific departures from these predictable components.

However, several statistical issues arise as a consequence of using space–time models. Possibly the most important of these is the increase of the sparseness in the counts. Even in pure spatial analyses that aggregate data at a certain spatial level over a given time period, counts can be small if working with small areas and/or rare diseases. Hence, the benefits of disaggregating over time and modeling in the time dimension with space–time interaction parameters need to be carefully calibrated. The aim of our study was to demonstrate that it is possible to exploit the rich output of space–time model fitting to gain interpretability without losing power.

Throughout this article, the epidemiologic context that we are considering is the analysis of the geographic and temporal variations of the risk of nonchromosomal congenital anomalies in England over a 16-year period. These variations can potentially be associated both with socioeconomic and environmental factors such as proximity to landfill sites (Elliott et al. 2001) and with heterogeneity in recording practices (Boyd et al. 2005), and there is great interest in examining whether any estimated spatial pattern is artifactual or occurs in a stable manner over time.

## Material and Methods

### Hierarchical space–time models for count data

The data consist of the observed number of congenital anomalies *Y_it_* and total number of births *n_it_* for area *i* = 1, … , *I* and year *t* = 1, … , *T*. Because the rate of congenital anomalies is approximately 1 per 1,000 births, a binomial model for the counts is more appropriate than the usual Poisson model. Without loss of generality, the space–time models that we will formulate will thus be within the binomial framework with logistic link. Extending these models to the Poisson case is straightforward. In the Bayesian hierarchical framework that we consider, the binomial likelihood of the data is the first level of the model, that is, for modeling the within-area variability of the counts conditional on unknown risk parameters. We give these parameters prior distributions at the second level of the model where we specify the space–time structure. The aim of the model is to characterize space–time patterns that are predictable from the data over the whole time period and to uncover any atypical departures from these patterns. We thus introduce different sets of parameters to represent, respectively, predictable global space–time structures and specific space–time interactions.

At the first level, we define a binomial model for the within-area variability of the counts:


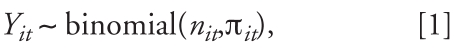


where π*_it_* is the risk of, say, congenital malformation in area *i* and year *t*. At the second level of the model, we split the risks π*_it_* on the logit scale into an overall risk α, main spatial effects λ*_i_*, main temporal effects ξ*_t_*, and space–time interaction terms ν*_it_*. We treat all these effects as random variables and give them prior distributions that specify how information can be borrowed across space or time in order to better capture the underlying structure of the risks.

The main spatial and temporal effects, λ*_I_* and ξ*_t_*, are the “predictable” parts, and we extend the spatial model introduced by Besag et al. (1991) to model their distribution. The spatial dependence is represented by means of a prescribed neighborhood graph that defines for each area *i* its set of neighbors (e.g., adjacent areas) denoted by δ*_i_*. Associated with this graph is an adjacency *I × I* matrix, say, **W** = (*w_jk_*), where *w_jk_* = 1 if we consider areas *j* and *k* adjacent, and 0 otherwise. Similarly, we define time neighbors simply by the two adjacent time points, with associated time adjacency matrix **Q**. The most commonly used parametric model to express spatial dependence is the conditional autoregressive model (CAR). Given an adjacency matrix **W**, this specifies the conditional distribution of a set of parameters μ*_i_* by


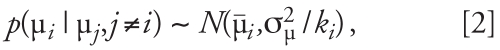


where σ_μ_^2^ is an unknown variance parameter, μ*_Ī_ =* ∑*_j__∈∂^i^_* μ*j* /*ki,* and *ki* is the number of neighbors of area *i*. Thus, the value of a parameter in one area is influenced by the average value of its neighbors, with additional variability quantified by a conditional variant σ_μ_^2^ /*k_i_*. We will use the notation μ ∼ CAR(**W**, σ_μ_^2^) to denote the conditional autoregressive process specified in [2], where μ is the vector (μ_1_, μ_2_, . . . , μ*_I_*)′.

This CAR model assumes a strong dependence and has only one free parameter linked to the conditional variance σ_μ_^2^. To increase flexibility, we use as spatial prior the sum of a CAR process and an unstructured exchangeable normal component with mean 0 and variance σ_λ_^2^. We will refer to this model as the convolution BYM model to acknowledge its introduction by Besag, York, and Mollié in 1991. It can be written in compact form as


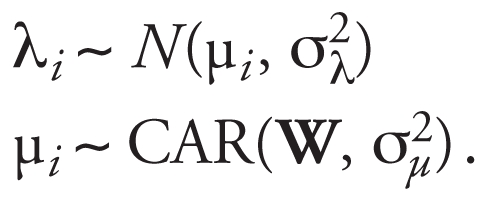


We have introduced the CAR and BYM model in the spatial context, but the definition is equally applicable in modeling temporal structure. The second level of our model is thus given by


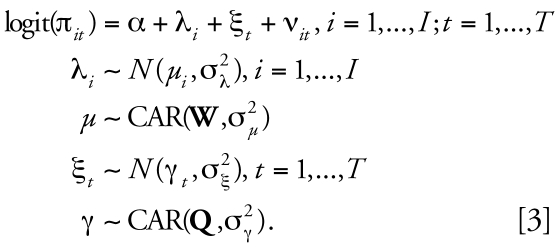


In Equation 3, besides separable spatial and temporal BYM structures for the logit risk, we have introduced space–time interactions parameters {ν*_it_*, *i* = 1, … *I ; t* = 1, … , *T*}, which capture any departure from predictable patterns based on the overall time trend and the overall spatial risk surface. These space–time interaction parameters are thus key for characterizing the stability of the underlying spatial patterns, with large fluctuations of (ν*_it_* , *t* = 1, … , *T* ) indicating instability of risk in area *i*. In the next section, we discuss in detail how to specify a prior distribution for the (ν*_it_* ), which helps to distinguish stable predictable patterns from atypical ones.

As in any Bayesian analysis, we define a third level of the model so that the variance parameters that are involved in the second-level equations (Equation 3) are themselves treated as unknown and given (hyper)prior distributions. We chose inverse gamma with parameters 0.5 and 0.0005, following Wakefield et al. (2000). To help identifiability of the parameters, we imposed sum-to-zero constraints on the vectors μ and γ.

### Characterizing patterns of space–time interactions

We must consider several issues when specifying a prior structure for the interactions {ν*_it_*, *i* = 1, … *I; t* = 1, … , *T* }. For non-infectious health outcomes, one would expect that the overall space and time components capture adequately most of the structure and that substantial space–time interactions are not common. Hence, some smoothing of the ν*_it_* parameters is necessary to ensure that we do not overparameterize the model and that noisy small space–time interaction parameters shrink toward zero. On the other hand, we also want to allow the possibility that a few areas have “true” departures from the overall stable model. This naturally leads us to choose a mixture model for the distribution of the ν*_it_* with two components: the first component models small ν*_it_* parameters that reflect only residual noise and are not of epidemiologic interest, whereas the second component captures “true” departures from the space and time main effects. Mixture models are typically used in Bayesian analysis when heterogeneity is suspected because they give a flexible prior structure that can be used for classification (Richardson and Green 1997). In our case, we are concerned with heterogeneity of the space–time interactions. Specifically, we consider


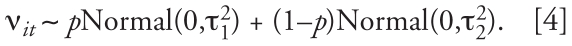


The prior for *p* is uniform on [0, 1], and we specify half-normal hyperprior distributions for the standard deviations τ*_k_*, *k* = 1, 2, to reflect that τ_1_ has to be small to effect shrink-age, whereas the prior for τ_2_ allows a large range of values for this parameter:


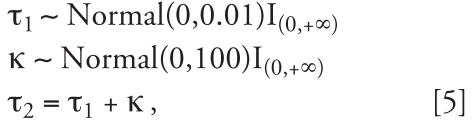


where I denotes the indicator function. The formulation in Equation 5 ensures that τ_2_ will always correspond to the variance of the second component in all simulations, hence avoiding label-switching problems.

As usual in the Bayesian mixture model formulation, we define latent allocation variables *z_it_* that take the value 0 or 1 if ν*_it_* comes from the Normal(0,τ_1_^2^) or the Normal(0,τ_2_^2^) components, respectively. We use these allocation variables to compute the posterior probabilities *p_it_* that each space–time interaction parameter ν*_it_* comes from, say, the second component: *p_it_* ≡ Pr(*z_it_* = 1|data). In turn, we employ these posterior probabilities to classify the areas into two subsets, *C*_1_ and *C*_2_, corresponding to “stable” and “unstable” risk patterns, respectively. Areas in *C*_1_ would be those showing small noninterpretable departures from main spatial and temporal effects. In contrast, areas classified in *C*_2_ will be those with higher levels of temporal variability in the risk, not due to chance. In the following, we consider two decision rules for defining “stable” and “unstable” risk patterns:

- Rule 1: An area *i* is in *C*_2_ (“unstable”) if *p_it_* > *p*_cut_ for at least one *t*, *t* = 1, . . . , *T*, where *p*_cut_ is a threshold probability to be defined.
- Rule 2: An area *i* is in *C*_2_ (“unstable”) if the average of the three highest posterior probabilities *p_it_* is greater than a threshold *p*_cut_.

Rule 1 is less restrictive than rule 2, which looks for more evidence of variability by averaging over three time points, which corresponds to roughly 20% of the 16 time points in our study. Neither rule makes any assumption about the specific shape of the pattern of the space–time interactions, but a variety of rules akin to rule 2 could be usefully constructed to look for variability in consecutive time points. We calibrate rules 1 and 2 in a simulation study and illustrate them in our case study.

### Congenital anomalies data

Here we consider the analysis of the geographic and temporal variation of risk of congenital anomalies in England between 1983 and 1998. We obtained data on live births and stillbirths from the births registry; we considered all nonchromosomal congenital anomalies combined [*International Classification of Diseases, 9th Revision*, codes 740–759 (World Health Organization 1975); *International Classification of Diseases, 10th Revision*, Q00–Q99 (World Health Organization 1993)] from the National Congenital Anomalies System. Both registers use post codes (~ 1.5 million post codes in England, 15 households on average) and are maintained by the Office for National Statistics. A copy of the data is held by the U.K. Small Area Health Statistics Unit (2008).

**Figure 1:**
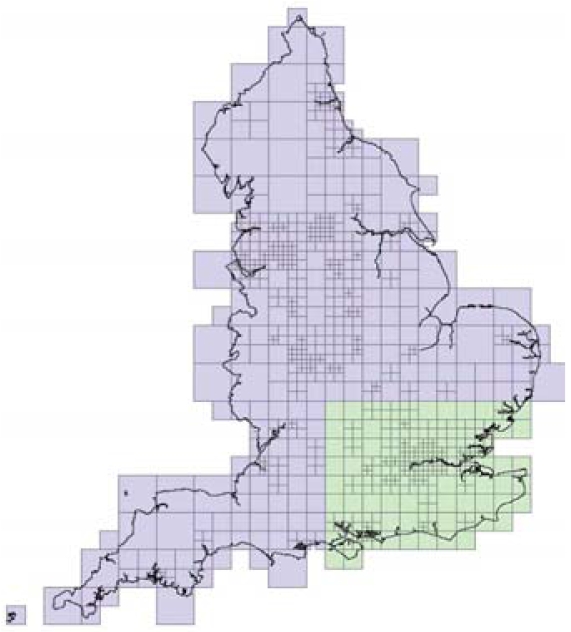
Grid of variable-size squares covering England (lilac), and squares considered for the simulation study (green).

We divided England into a grid of around 5,500 squares of 5 × 5 km based on the U.K. National Grid. We compiled all data in a geographic information system, based on a conformal projection (Universal Transverse Mercator), with a notional resolution of 1 m. For each grid square, we estimated the number of births and the number of congenital anomalies from the residential post code locations. Because this led to very sparse data for some years and squares, we decided to aggregate some of the squares, in order to have no more than a 4- to 5-fold interquartile range in the number of births per square over England. Thus, the geography used in our analysis divides England into a grid of 970 variable-size squares (Figure 1). We aggregated the annual number of both congenital anomalies and births for each square in the new grid. Table 1 gives a descriptive summary of the data.

### Data generation

Besides the case study, we evaluate the performance of our space–time model formulation and, in particular, the classification of areas into “stable” and “unstable” in a comprehensive set of simulations. In order to be realistic in terms of hypothesized spatial variability of risk and number of births, we based our simulations on the congenital malformation setup but with a reduced set of 309 areas corresponding to the southeast of England in order to ease the computational burden (Figure 1).

To be precise, we use the posterior medians of the spatial and temporal main effects, λ_i_^*^ [range, on the odds ratio (OR) scale, between 0.3 and 2.1] and ξ_i_^*^ (from 0.62 to 1.79 on the OR scale), derived from fitting the model in Equation 3 to the congenital malformation data [as well as α* = log(0.01)] to generate the predictable spatial and temporal patterns for the data replications in our simulation study. For a subset of squares *M* we also add space–time interaction with different variances. Specifically, we define the set of simulated risks as


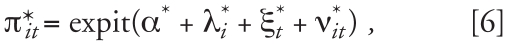


where expit(*x*) = exp(*x*)/[1 + exp(*x*)], and


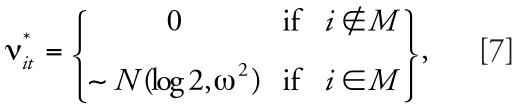


where *M* is a subset of squares for which the risk is modified and perturbed by noise. We consider three different cases for the amount of noise: a reference case with no noise (ω^2^ = 0), a medium-variance scenario with a medium amount of noise [ω^2^ = (0.5)^2^], and a high-variance scenario with a high level of noise

**Figure 2:**
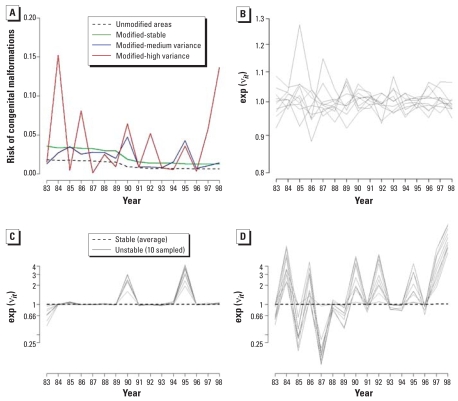
(*A*) Risk profiles of the unmodified squares compared with those modified under each variance case, represented on the probability scale. (*B*–*D*) ORs on the logarithmic scale (risk pattern profiles, *M*_8_ case): (*B*) 10 most “atypical” estimated profiles in the reference case (expanded scale); (*C* and *D*) estimated risk profiles for 10 randomly selected areas classified as “atypical” using rule 1 in the medium-variance (*C*) and high-variance (*D*) cases for the *M*_8_ scenario.

[ω^2^ = (1.5)^2^]. We generated values for ν_it_^*^, *i* ∈*M* just once. Figure 2A displays profiles of the risk patterns (π_it_^*^) for each variance case. Between the reference case (green line) and the medium case (blue line), the risks vary about 2-fold. We do not expect the variability generated for the high-variance case (red line) to be realistic; rather, we use this case as a benchmark.

Using the simulated risks π_it_^*^, we generated 50 data replications, one for each variance type scenario. This is done separately for each square, using a multinomial distribution, based on the total number of observed cases, *Y_i_* =∑*_t_*_=1_*^T^* *Y_it_*, in each square. This ensures that the total number of cases in the replication was the same as in the original data. In other words, we generated the *r*th data replication for area *i*, **Y**^(^*^r^*^)^ = (*Y_i_*_1_^(^*^r^*^)^,…,*Y*_iT_^(^*^r^*^)^)′, from


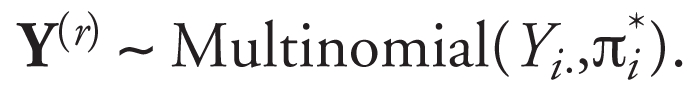


Note that because the aggregated number of cases in each square is the same for all replications, a pure spatial model fitted to the time-aggregated data will give exactly the same results regardless of the variance scenario.

Finally, besides the different variance cases for the variability of the space–time interactions, we also consider three different scenarios for the proportion of modified squares. Indeed, the fitting of the model in Equation 3 and, in particular, its ability to separate effectively the predictable part from the space–time interactions will be influenced by the overall number of modified (i.e., unstable) squares. Thus, we consider the three cases of 20%, 8%, and 1% of modified squares in *M*, which we denote hereafter as *M*_20_, *M*_8_, *M*_1_, respectively. The exact numbers of squares were |*M*_20_| = 59, |*M*_8_| = 26, and |*M*_1_| = 3 [Supplemental Material, Figure 1 (http://www.ehponline.org/members/2008/10814/suppl.pdf), shows the squares selected for each scenario].

## Results

### Model implementation and convergence issues

Bayesian inference is based on the joint distribution of all parameters (e.g., λ*_i_*, ξ*_t_*, ν*_it_*) given the data. In our case, this joint distribution is intractable analytically, so instead we simulated it using the framework of Markov chain Monte Carlo (MCMC) algorithms (Gilks et al. 1996) that is now commonly used for Bayesian inference in a wide variety of applications. We used the free software WinBUGS (Spiegelhalter et al. 2003), based on MCMC algorithms, to implement all models [Supplemental Material, Appendix 1 (http://www.ehponline.org/members/2008/10814/suppl.pdf), gives the WinBUGS code]. For the simulation study, we based the results on a thinned (every 10th) sample of 2,000 observations from the posterior distribution of the parameters, after discarding the first 10,000 as burn-in. We used a longer run for the case study (50,000 iterations after a 150,000 burn-in).

It is well known that mixture models are difficult to estimate and that, for example, multimodal likelihood and label switching problems can occur (Richardson and Green 1997). Here, we circumvent label switching issues by the constraint τ_2_ = τ_1_ + κ, κ > 0. One issue is whether a data set has enough information for us to estimate a two-component mixture for the variance of the interaction parameters. In any application of our model to real data, we expect sizable residual variability once we fit the predictable part, because we deliberately kept the structure of the predictable part simple. Hence, the space–time interaction parameters will absorb this residual variability, and there will be enough heterogeneity to support the estimation of the two mixture components for their variance. Note that when defining the prior for τ_1_, consideration should be given to the size of the residual variability. In our reference simulation scenario, however, the estimation of a two-component mixture is artificial, and we expect substantial overlap between the two components. This is confirmed by the large posterior variability of the mixture parameters in the reference case (Table 2).

We judged convergence of chains on visual checks by running two chains with different starting values. We ran chains long enough that the ratio of the Monte Carlo error to the posterior standard deviation was small, as is usually recommended (Roberts 1996), roughly < 5%. Supplemental Material, Figure 2 (http://www.ehponline.org/members/2008/10814/suppl.pdf), displays graphs of the posterior densities of the mixture parameters in the case study for two separate chains and illustrates how two chains with widely different starting points led to the same posterior distribution. Supplemental Material, Figures 3 and 4 (http://www.ehponline.org/members/2008/10814/suppl.pdf), show the time series plots of the last 199,500 simulations without and with thinning, respectively, for each of the parameters in the mixture component.

### Estimation of spatial patterns

We first want to show that in cases where the spatial structure is stable, the additional complexity of the space–time analysis does not perturb the estimation of the stable spatial patterns. Indeed, in cases with a stable pattern, introducing space–time parameters unnecessarily overparameterizes the model and hence could lead to a loss of precision. We estimate a pure spatial model on the time-aggregated counts *Y_i_* where we replace [3] with


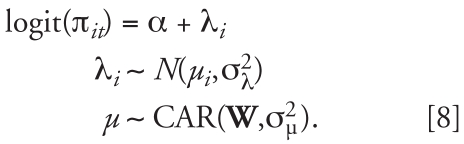


For this comparison, we use the reference case of the *M*_20_ scenario described in the preceding section. Comparison of the estimated spatial risks between the spatial-only model [8] and the spatiotemporal model [3] reveals that the estimated spatial risks λ*_i_* are almost identical between the pure spatial model and the space–time model (correlation = 0.99) despite the increase in the sparseness and the inclusion of many more parameters in the space–time analysis.

Besides the estimation of the λ*_i_*, it is also of interest to examine the posterior probabilities Pr[exp(λ*_i_*) > 1 | data], because a previous study (Richardson et al. 2004) has shown that these can be used to pinpoint areas of elevated risks. In particular, Richardson et al. (2004) suggest that a decision rule that thesholds these posterior probabilities > 80% gives a good compromise between sensitivity and specificity for detecting areas of increased risks. Comparison of the posterior probabilities estimated with the pure spatial versus the space–time model shows little discrepancy (correlation = 0.99) and hence no loss of power when using a space–time model instead of a pure spatial model. Numbers confirm this: Of the 147 areas with true spatial excess risk of at least 10% (including both modified and unmodified areas), 110 areas would be detected by such a rule in the pure spatial case (same number in all replications by construction), and 108 on average (over all replications) when using the space–time model. Therefore, the sensitivity of this rule is around 74%, which is adequate and similar for both models.

### Estimation of the space–time interactions

**Figure 3:**
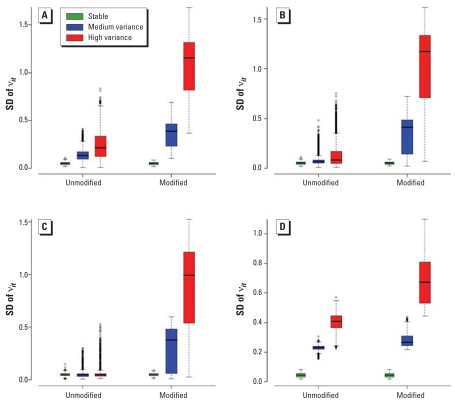
Box plots of the posterior median of the empirical standard deviations of the space time interactions when we modeled ν*_it_* as a mixture of two normals: *M*_20_ (*A*), *M*_8_ (*B*), and *M*_1_ (*C*) scenarios. (*D*) Box plot of the empirical standard deviations using an exchangeable normal model for the space–time interactions.

As a first check on the performance of our space–time model with mixture prior on the interactions, we computed the empirical standard deviation of the ν*_it_* :


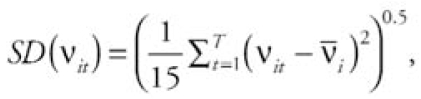


where ν*_Ī_* is the average of the ν*_it_* over the 16 time points. Figure 3A–C shows box plots of SD(ν*_it_* ) for the three variance cases (reference, medium, and high) and the different scenarios *M*_20_, *M*_8_, and *M*_1_.

The reference case where all patterns are stable shows, as expected, that ν*_it_* values are small and identical between the modified and unmodified areas in all scenarios. Comparison between the medium-variance box plots (blue) and the high-variance box plots (red) for the modified areas shows that the model captures well the increased variability of the space–time interactions, irrespective of the number of modified areas. Comparison among the *M*_20_, *M*_8_, and *M*_1_ scenarios shows that the increasing number of modified areas has some influence on the overall fit of the model and hence the space–time patterns in the unmodified areas. Indeed, in the *M*_1_ scenario, the SD(ν*_it_*) values of the unmodified areas are small and fairly similar for the reference, medium-, and high-variance cases. As the number of modified areas increases, SD(ν*_it_*) for the unmodified areas also increases somewhat, especially for the high-variance case. We expect this because the large fraction (20%) of unstable areas in this scenario renders the “stable” pattern less typical and blurs the distinction between stable and unstable cases.

We also display in Figure 3D the equivalent box plots that would be obtained in the *M*_20_ case if instead of a mixture model [4] for the space–time interactions, we had simply assumed that these interactions came from an exchangeable distribution with common variance, ν*_it_* ~ Normal(0, τ^2^). From this plot, we can clearly see that an inappropriate exchangeable assumption leads to a substantial blur between the estimated variability of the interaction parameters of modified and unmodified areas, and that interpreting any patterns of the variability of the interactions as corresponding to “true variability” would be difficult. Hence, the specification of the mixture model (Equation 4) is key for the good performance of our approach.

Table 2 summarizes the posterior estimates of the mixture parameters for all three proportion scenarios and variance cases. The parameter *p* estimates the proportion of space–time “pixels” showing departure from main effects. Because of the design of our simulation setup, we would roughly expect 1 – *p* to reflect the proportion of modified areas, that is, 20%, 8%, and 1%, respectively. Both the medium- and high-variance cases estimate this proportion reasonably well, with relatively narrow 95% credibility intervals, the only exception being the medium case in the *M*_20_ scenario. As expected, the values of τ_1_ are quite small in all variance scenarios and proportion cases, because the predictable part is the true model for the unmodified areas. The high-variance scenario estimates the values of τ_2_ well: The true value 1.5 is within the average 95% credibility interval. However, the medium-variance case overestimates τ_2_ somewhat (estimated value is around 0.7, whereas the true value is 0.5). This could be due to the prior on τ_1_ being a little too restrictive and thus biasing some interactions toward the second component.

In the reference case, where there is no true variability over time, the mixture artificially separates ν*_it_* into two groups, which would be misleading to interpret. However, the difference between τ_1_ and τ_2_ is quite small, thus indicating that the second component quantifies only a modest increased variability of the risks over time. Moreover, the credibility intervals for τ_1_ and *p* are large, pointing to lack of identifiability, as would be expected in this case (see “Model implementation and convergence issues,” above). In the next section, we discuss other indicators that would suggest that, in this case, the second component is indeed not capturing real evidence of variability of the risks.

To challenge the mixture model further, we also considered another version of the *M*_8_ scenario that included some additional background noise (of the order of 10% on the relative risk scale) for the unmodified squares in the generation of the fake risks π_it_^*^. In particular, in Equation 6 we introduced space–time interactions generated not according to Equation 7 but by the following:


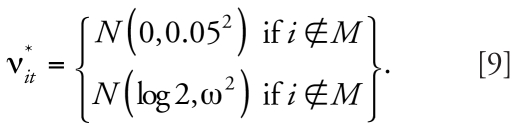


Results were virtually the same.

### Performance of the classification rules

**Figure 4:**
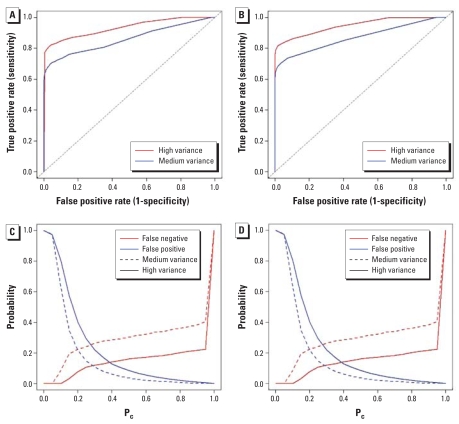
ROC curves (*A* and *B*) and curves of false-negative and false-positive rates (*C* and *D*) associated with rule 1 (*A* and *C*) and rule 2 (*B* and *D*) for the *M*_8_ scenario.

Because we are in a simulation setup with “truly” stable and unstable areas, it is possible to assess the sensitivity and specificity of the rules 1 and 2 for classifying areas as “unstable.” Figure 4, A and B, shows average receiver-operating characteristic (ROC) curves (sensitivity vs. 1 – specificity for different cutoff values *p*_cut_) for each variance case for the *M*_8_ scenario corresponding to rules 1 and 2, respectively. As expected, the sensitivity is higher in both rules for the easier high-variance case (red line).

For rule 1, one can see that for a specificity of 10%, the power is around 70% in both cases, which makes this rule useful. To better interpret the ROC curves, Figure 4, C and D, shows complementary plots of the observed fraction of false positives and of false-negative versus cutoff values. In the realistic medium-variance case, the lines cross around *p*_cut_ = 0.2 for the *M*_8_ scenario (Figure 4), and *p*_cut_ = 0.4 for the *M*_20_ case (not shown). These cutoff values offer a compromise between the two types of errors, which are balanced at around 20%. In our context, however, the two types of errors are not symmetric, and it is typically more important to achieve high specificity. If we wish to control the false-positive rate at around 10%, say, then we should consider higher values for the cutoff. A value of *p*_cut_ = 0.5 guarantees good specificity in both scenarios, with around 20% false-negative rates.

Performance of rule 2 is very similar to that of rule 1 (Figure 4). The false-positive and false-negative curves cross at *p*_cut_ = 0.2 for both *M*_8_ (Figure 4) and *M*_20_ (not shown) scenarios in the medium-variance case. By construction, rule 2 is more conservative than rule 1 and will require somewhat lower thresholds for the same specificity.

Once we classify the areas, we can interpret the variability by looking at the time profiles of the estimated interactions. Figure 2C,D shows a sample of profiles of the space–time interactions estimated for the areas classified as “unstable” according to rule 1 and a cutoff value *p*_cut_ = 0.5. Comparing these profiles with the simulated pattern (Figure 2A) shows that the simulated pattern is well recovered. Note that, in general, one would not expect, as in our simulated case, the same time profile of variability for all areas. Because this does not influence the classification results (the ν*_it_* are modeled independently over *i*), we did this in our simulation setup to display the results better. In our case study, we show how exploratory clustering of the time patterns for the areas declared “unstable” is necessary to confer additional interpretability.

Figure 2B shows a sample of time profiles for the reference case. Using rule 1 and a cutoff of 0.5, on average, over the 50 replications no areas would be classified as “unstable.” Looking at the profiles of the 10 areas with highest space–time variability among the replications with highest values of SD(ν*_it_*), we can see that even in these “extreme” realizations the variability is small, especially compared with the medium- and high-variance cases. Notice the different scale of the y-axis in the plot of the reference case (Figure 2B) compared with the other two scenarios (Figure 2C,D). In fact, if plotted in the same scale, the space–time profiles are hardly distinguishable from the stable profile of the medium- and high-variance cases.

Finally, we investigated the combination of rule 1 with the rule Pr[exp(λ*_i_*) > 1 | data]. Of the 250 unmodified areas in the *M*_20_ scenario, 91 had λ_i_^*^> 1.1; of those, 84 and 85 had Pr[exp(λ*_i_*) > 1] > 0.8 in the medium- and high-variance cases, respectively, and we classified all of them as stable using rule 1. Analogously, of the 283 unmodified squares in *M*_8_, 98 had λ_i_^*^>1.1; of those, 94 and 93 had Pr[exp(λ*_i_*) > 1] > 0.8 in the medium- and high-variance cases, respectively, and again, we classified all of them as stable by rule 1. This shows that our modeling approach combined with appropriate classification rules can effectively identify the areas where elevated risk is occurring in a stable way.

### Case study: risk of nonchromosomal congenital anomalies in England

**Figure 5:**
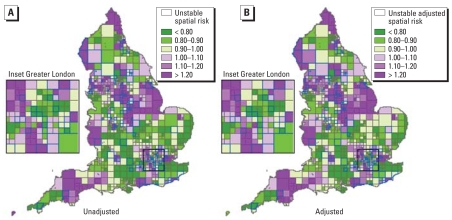
Maps of unadjusted (*A*) and adjusted (*B*) spatial risks for the case study. Overimposed with blue borders are the areas classified as “atypical” using rule 1.

We estimated the hierarchical space–time model (Equations 3 and 4) on the congenital malformation data. Figure 5A shows the estimated spatial pattern. There is evidence of spatial heterogeneity of the relative risks, with higher values estimated for regions in the north, northeast, and northwest, some central areas, and the southwest. The densely populated Greater London area (inset) also has squares with high risks. Maternal age and deprivation are known risk factors for some nonchromosomal congenital anomalies. They likely contribute to the geographic pattern of the overall risk shown in Figure 5A. To partially explore this, for each square we calculated the Carstairs score, a well-known measure of socioeconomic deprivation in the United Kingdom (Carstairs and Morris 1989), and included it in the model as a continuous covariate. The OR was significant (OR = 1.01; 95% credibillity interval, 1.01–1.02), although the adjusted spatial pattern in Figure 5B looks very much like the unadjusted one (correlation = 0.98).

**Figure 6:**
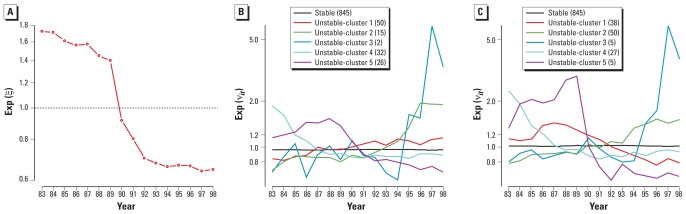
Estimated main time trend in the case study (*A*), and estimated risk profiles of the clusters of “atypical” areas according to rules 1 (*B*) and 2 (*C*) in the case study.

The temporal trend for nonchromosomal congenital anomalies (Figure 6A) shows a clear drop in 1990 and subsequent decrease in 1991 and 1992, after which the time trend stabilizes. In 1990, some minor anomalies were reclassified and excluded from the combined congenital anomalies, hence the level shift [for details on the *International Classification of Diseases* codes excluded, see Office for Population Censuses and Surveys (1995)].

Table 2 (bottom row) shows posterior medians and 95% credibility intervals for the mixture parameters in the case study. In contrast to the simulation results, the posterior median of the standard deviation of the first component, τ_1_, is larger. This is not surprising; in our simulation setup, the risk in the unmodified areas was totally predictable from the separable space and time components, so the first component was just capturing noise created by the overparameterization. In contrast, for any real data set, some departure from the predictable pattern will be observed for almost all areas, the extent of which is then modeled by the mixture model. The first mixture component will thus summarize a small lack of fit of π*_it_* from the predicted risk built by λ*_i_* and ξ*_t_*, which is not worthy of further investigation. There was no indication of lack of convergence in the visual checks [see Supplemental Material, Figure 2 (http://www.ehponline.org/members/2008/10814/suppl.pdf)], and the credibility intervals for the mixture parameters were relatively narrow, indicating that there is sufficient information to estimate the mixture model. This is in keeping with our simulation setup, where with a similar range of denominator counts and variability of the space–time interactions patterns, we showed that our method has good operational characteristics.

Using rule 1 and a cutoff of 0.5, we classified 125 of the 970 (13%) variable-size grid squares as exhibiting some instability. Blue borders in Figure 5A highlight these squares. They do not appear to be specifically linked to a particular region. The proportion of squares classified as “unstable” is close to the estimation of (1 – *p*), which would be 16% and represents the number of space–time “pixels” in the second component of the mixture.

To interpret the variability shown in the squares classified as “unstable,” it is useful to attempt to cluster the time profiles of the interactions. Using simple hierarchical clustering (Mardia et al. 1979) on these time profiles, we found five main profile patterns (Figure 6B,C). Four subgroups exhibit smoothlike trends (increasing or decreasing) over time, indicating that the interactions terms are used to “adjust” these areas to the general time trend. Indeed, the implementation of the recommended reclassification of some minor anomalies did not happen at the same speed over the United Kingdom, and thus we expect shift from the overall time trends. Some areas had higher rates before 1991 and decreased more steeply than the average (cluster 5), whereas for others the difference with the average is only marked before (cluster 4) or after (cluster 2) the change to the minor anomalies classification. Besides these groups, one small subgroup (two squares in cluster 3) shows a sudden high peak in the year 1997 (blue line). This unexpected and large time variation in the risk could correspond to an abrupt change of local recording practice or to a real excess of cases, either situation warranting further investigation. In fact, we looked further into the data for these two squares and found an important increase of reported kidney malformations in that year. We contacted the local register covering those two squares, and interestingly, they traced back the increase to artifactual changes in recording practices.

We found a close correspondence between the areas classified as “unstable” by rule 1 and by rule 2. The top 125 areas that would be considered “unstable” by rule 2 (ranked by their score for rule 2) would contain 104 of the areas similarly classified by rule 1. This leads, of course, to similar clusters of time profiles (Figure 6C).

As mentioned above, the inclusion of the Carstairs index had some influence in the spatial pattern, but it did not affect the main time trend and had only a marginal effect on the space–time interactions. Using rule 1, we found 119 “unstable” areas, 117 of which were among the 125 found in the unadjusted case. These results can be expected, because the Carstairs score was not time dependent; that is, we included it as a spatial-only covariate with one single value for each area.

## Discussion

The use of Bayesian hierarchical spatial models has become widespread in disease-mapping and ecological studies of health–environment associations. In most of the studies carried out, the data are typically aggregated over an extensive time period, typically more than a decade. Extension of hierarchical spatial models to space–time modeling of one or several diseases has been discussed by a number of authors (Bernardinelli et al. 1995; Knorr-Held 2000; Knorr-Held and Besag 1998; Richardson et al. 2006; Waller et al. 1997). The purpose of these authors was to propose and investigate the fit of a variety of space–time model formulations. In the present article, we build on these extensions, but our purpose is different: We aim to distinguish, in a statistically informed way, “atypical” areas from areas where the risk is “predictable” by a simple combination of overall spatial pattern and time trends. Our purpose is *a*) to strengthen the interpretation of the geographic patterns of risk that are “sustained” over time and *b*) to pinpoint “atypical” or “unstable” areas showing evidence of unusual variability in the time pattern of the risk.

The epidemiologic interpretation of “predictable” versus “atypical” patterns has to be done with respect to the health outcome investigated. In the case of a disease where short-latency effects are plausible, such as congenital malformations, unusual variability, especially excess risk, is important to investigate further because it may point to the emergence of a new environmental hazard. On the other hand, stable spatial patterns are more likely to be linked to recurrent sociodemographic and lifestyle risk factors, as well as to prolonged environmental hazards. For other health outcomes, “atypical” risk patterns over time may point toward a local change in recording practice or health care. For example, a sudden increase or decrease in the risk of “avoidable deaths” in some areas might give an early warning that health care is deteriorating or improving in those areas. Hence, routine evaluation of space–time patterns could be built into a surveillance system.

Because of the complex dependence patterns over space and over time of the occurrence of many chronic health outcomes, and the inherent large stochastic variability due to rare events, estimating separately time trends in each area will not be efficient because it will be difficult to establish a baseline pattern separately for each area. In our Bayesian approach, we use the power of hierarchical modeling to borrow information over space and time in order to estimate typical predictable patterns for each area. We further strengthen the inference by using a joint mixture model for the space–time interactions, which again borrows information across all the time points and areas to improve inference, while at the same time explicitly recognizing their heterogeneity. The mixture model uses prior knowledge to specify meaningful priors. In particular, for the sake of identifiability, sensible prior assumptions are important for the variance of the component of the mixture that captures the idiosyncratic space–time interactions (in our formulation, the first component) so that the mixture achieves a meaningful separation between small noninterpretable and “truly” large space–time interactions.

The space–time hierarchical model that we have formulated is easily implemented by freely available WinBUGS software and thus provides a useful tool for health–environment investigations and health-practice surveillance. We tested the performance of this novel formulation in several simulation scenarios and demonstrated that by postprocessing the rich output of the Bayesian space–time model, we can build classification rules that have good operational characteristics to detect “atypical” areas. We based our simulations on realistic scenarios inspired by our case study. In this way, we strengthened the interpretation of the space–time interaction patterns found in the case study. Our simulations also showed that, with respect to estimating the overall spatial pattern, using a more sophisticated model that includes a time element and space–time interactions does not damage the estimation of the pure spatial pattern. Rather, it helps to characterize spatial excess risks that are stable over time. We performed our simulations within the framework of binomial variability, with expected median number of events per area per year between 3 and 10. Extension of our simulation setup to the Poisson case and to smaller numbers of expected events per area would be interesting and will be considered in the future.

Clearly, once the model achieves a reasonable classification of areas, it is of interest to explore further the time pattern of the risks in “atypical” areas. In our simulation setup, we deliberately chose not to introduce any specific time patterns, for example, step changes that could signal the occurrence of a new environmental hazard. If detection of such situations were of particular epidemiologic interest, modified rules to declare areas “atypical” could easily be defined and calibrated. Moreover, instead of using rules based on *p_it_*, which are marginal posterior probabilities for each *i*, *t*, we could investigate how to exploit the joint distribution of the allocations **z***_i_* = (*z_i_*_1_, . . . , *z_iT_*)′.

The choice of the variable-size grid squares might, of course, have had an effect on the results. In fact, this is the so-called modifiable areal unit problem, “a problem arising from the imposition of artificial unit of spatial reporting on continuous geographic phenomenon resulting in the generation of artificial spatial patterns,” as defined by Heywood et al. (1998). There is always a balance between geographic resolution and “detectable” size of effects. Strong patterns are hard to mask by the specific aggregation chosen, whereas small effects may be more sensitive to aggregation. The size of the expected counts is relevant, and we used those to select our grid squares in order to keep sparseness under control.

Inclusion of covariates in our model is mathematically straightforward, but less so from an epidemiologic point of view. Covariates may be purely spatial (i.e., one single value for each area, constant throughout time), or purely temporal (one single value for each time point, the same for all areas) or spatiotemporal. Spatial-only covariates may help explain the spatial pattern, but should not affect the temporal trend or the interaction.

The interpretation of the geographic pattern is affected by including spatial-only covariates in the model, because it becomes the spatial pattern of risk after adjustment for the main spatial effects of covariate. Analogously, temporal-only variables could explain the main time trend but would not affect the spatial pattern or the space–time interaction. More delicate might be the inclusion of covariates that vary in both space and time. They could affect the main spatial and temporal effects, as well as the space–time interactions, thus complicating interpretation of the results.

Further time-varying or space–varying coefficients could be considered, allowing for different effects of covariates in time or space, thus increasing considerably the flexibility of the models but at the same time creating difficult model choice issues. We believe this is an interesting area to pursue in the future.

In the case study, we also fitted our model with the Carstairs score, a well-known area-based index of deprivation in the United Kingdom. It is based on four census variables and can therefore be computed for 1981, 1991, and 2001. Internal analyses at the U.K. Small Area Health Statistics Unit showed that the three years are strongly correlated (correlations > 0.9), thus suggesting that material deprivation is rather stable over time, at least between 1981 and 2001, and hence over the period considered in our study. For this reason, we decided to include this variable in the model as spatial only.

In our analysis of congenital malformations in England, we employed standard hierarchical clustering of the time profiles of “atypical” squares to uncover interesting patterns. How to improve classification rules as well as tailoring statistical models to cluster the time profiles efficiently is an interesting extension of our approach that we plan to include in future publications.

In conclusion, here we introduced a novel class of space–time models and demonstrated how epidemiologic interpretation of risk patterns is considerably strengthened by the inclusion of the time dimension.

## Correction

In the manuscript originally published online, Figure 4C was incorrect; it has been corrected here.

## Figures and Tables

**Table 1:** Descriptive summary of the case study data: distribution of cases and denominator counts across areas.

Year	5th percentile	1st quartile	Median	Mean	3rd quartile	95th percentile
Congenital anomalies						
1983	0	3	9	13.67	18	43
1984	0	4	9	13.67	18	40
1985	0	3	9	12.99	18	41
1986	0	3	9	12.85	17	40.6
1987	0	4	9	13.39	18	41.6
1988	0	3	9	12.75	17	41
1989	0	3	8	12.21	16	39
1990	0	2	6	8.007	11	25.6
1991	0	2	5	6.916	10	21
1992	0	2	4	5.994	8	18
1993	0	1	4	5.638	8	18
1994	0	1	4	5.520	8	17
1995	0	1	4	5.451	7	17
1996	0	1	4	5.485	7	17
1997	0	1	4	5.305	7	16
1998	0	1	3	5.249	7	18
Births and stillbirths						
1983	43	220	503	615.6	896	1420.2
1984	43	222	503	623.2	906	1435.6
1985	46	233	522	642.7	929	1493.6
1986	44	233	528	647.5	945	1,504
1987	46.8	245	542	667.3	979	1522.6
1988	46	253	560	678.4	1,005	1559.4
1989	49	251	552	673.5	981	1480.6
1990	46	252	562	691.2	1,018	1558.2
1991	46	253	558	684.9	1,001	1528.4
1992	45	252	553	678.4	984	1537.4
1993	47	243	544	660.3	962	1492.8
1994	46	241	537	654.1	945	1,475
1995	43	237	530	638	915	1444.8
1996	45.8	241	528	639.2	906	1445.2
1997	46	236	522	632.7	891	1446.4
1998	45.4	237	524	626.5	886	1452.8

**Table 2:** Posterior medians (95% credibility intervals) of the standard deviations of the two mixture components and the mixing proportion *p* (all averaged over the 50 replications), obtained under each scenario.

Model	Scenario	τ_1_	τ_2_	*p*
*M*_20_	Reference	0.04 (0.02–0.06)	0.16 (0.05–3.50)	0.75 (0.36–0.89)
	Medium	0.05 (0.04–0.07)	0.72 (0.63–0.82)	0.89 (0.85–0.91)
	High	0.04 (0.02–0.06)	1.39 (1.27–1.52)	0.81 (0.79–0.83)
*M*_8_	Reference	0.04 (0.02–0.06)	0.10 (0.05–2.76)	0.76 (0.36–0.91)
	Medium	0.04 (0.03–0.07)	0.75 (0.64–0.91)	0.94 (0.92–0.96)
	High	0.04 (0.02–0.06)	1.43 (1.27–1.63)	0.92 (0.90–0.93)
*M*_1_	Reference	0.03 (0.02–0.06)	0.10 (0.05–2.15)	0.73 (0.35–0.89)
	Medium	0.04 (0.03–0.06)	0.70 (0.46–1.30)	0.99 (0.97–1.00)
	High	0.05 (0.03–0.06)	1.32 (0.96–1.95)	0.99 (0.98–0.99)
Case study		0.31 (0.25–0.34)	0.63 (0.49–1.00)	0.88 (0.64–0.98)
