# Correction: Use of Space–Time Models to Investigate the Stability of Patterns of Disease

**DOI:** 10.1289/ehp.10814-erratum

**Published:** 2008-04-25

In the manuscript originally published online, Figure 4C was incorrect; it has been corrected here.

